# Habitat-specific differences in plasticity of foliar *δ*^13^C in temperate steppe grasses

**DOI:** 10.1002/ece3.970

**Published:** 2014-02-12

**Authors:** Yanjie Liu, Lirong Zhang, Haishan Niu, Yue Sun, Xingliang Xu

**Affiliations:** 1 College of Resources and Environment, University of Chinese Academy of SciencesNO. 19-A Yuquan Road, Shijingshan District, Beijing, 100049, China; 2 Key Laboratory and Ecosystem Network Observation and Modelling, Institute of Geographic Sciences and Natural Resources Research, Chinese Academy of SciencesNO.11-A Datun Road, Chaoyang District, Beijing, 100101, China

**Keywords:** Environmental variation, plasticity, species habitat, stable carbon isotope, temperate steppe, temporal variation

## Abstract

A decrease in foliar *δ*^13^C with increasing precipitation is a common tendency in steppe plants. However, the rate of decrease has been reported to differ between different species or populations. We here hypothesized that plant populations in the same habitat of temperate steppes may not differ in foliar *δ*^13^C response patterns to precipitation, but could differ in the levels of plasticity of foliar *δ*^13^C across different habitats. In order to test this hypothesis, we conducted controlled watering experiments in northeast China at five sites along a west–east transect at latitude 44°N, which show substantial interannual fluctuations and intra-annual changes in precipitation among them. In 2001, watering treatment (six levels, three replicates) was assigned to 18 plots at each site. The responses of foliar *δ*^13^C to precipitation (i.e., the sum of watering and rainfall) were determined in populations of several grass species that were common across all sites. Although similar linear regression slopes were observed for populations of different species growing at the same site, significantly different slopes were obtained for populations of the same species growing at different sites. Further, the slope of the line progressively decreased from Site I to Site V for all species in this study. These results suggest habitat-specific differences in plasticity of foliar *δ*^13^C in temperate steppe grasses. This indicates that species' *δ*^13^C response to precipitation is conservative at the same site due to their long-term acclimation, but the mechanism responsible behind this needs further investigations.

## Introduction

Phenotypic plasticity, the ability of an organism to alter its form or function in response to changes in environmental conditions, is believed to be an advantageous evolutionary response to environmental heterogeneity (Bradshaw 1965, 1973; Schlichting 1986; Silim et al. 2001). The level of plasticity is defined as the degree to which a trait value changes in response to a change in the environment (Liefting and Ellers 2008). To maximize their fitness, species that grow in an unpredictable or variable habitat are expected to have a high potential to acclimate and a high degree of plasticity (Bazzaz 1991; Brakefield et al. 1996; Agrawal 2001; Yeh and Price 2004; Richards et al. 2006).

The stable carbon isotope composition (*δ*^13^C) of plant tissues is related largely to the temporally averaged ratio of the concentration of intercellular to atmospheric CO_2_, *c*_*i*_*/c*_*a*_, which is the result of the balance between stomatal conductance and photosynthesis rate (Farquhar et al. 1982, 1989). It has been widely used as an indicator of intrinsic water-use efficiency (WUE) in ecological studies (Silim et al. 2001; Wang et al. 2012). As factors that affect either of these two processes – stomatal conductance and photosynthesis – also have effects on WUE, WUE is considered an integrated measure of physiological status and environmental conditions (Farquhar et al. 1989).

Water availability is an important factor that influences plant growth (McConnaughay and Coleman 1999; Poorter and Nagel 2000), particularly in the temperate steppes of Inner Mongolia, China. This study investigated the plasticity in foliar *δ*^13^C (WUE) in several plants species at large spatial scales in Inner Mongolia, China. Extensive evidence indicates that foliar *δ*^13^C decreases with moisture increases in plants (Schulze et al. 2006; Luo et al. 2009; Diefendorf et al. 2010; Prentice et al. 2011; Wang et al. 2012). Plasticity could contribute to this pattern (Corcuera et al. 2010). However, some species do not show a similar pattern at large spatial scales (Prentice et al. 2011), although their foliar *δ*^13^C decreases over time with increasing precipitation in the same habitat (Liu et al. 2013). Therefore, plant populations or species coexisting in the same habitat of temperate steppes might not show differences in foliar *δ*^13^C response patterns to precipitation, and selection for differences in patterns of plasticity might arise elsewhere in species distribution where they experience different environmental conditions, that is, differences in the levels of plasticity in foliar *δ*^13^C across different habitats.

Herein, we provide evidence for this above-mentioned prediction on the basis of five controlled watering experiments for several species along a precipitation gradient in the temperate steppes of China. Specifically, the following two questions are addressed: (I) Do all the species under different watering conditions (precipitation) in the same experimental site show similar levels of plasticity in foliar *δ*^13^C? (II) Are the levels of plasticity in foliar *δ*^13^C different among different sites; if so, which show higher plasticity?

## Materials and Methods

### Study location

**Figure 1:**
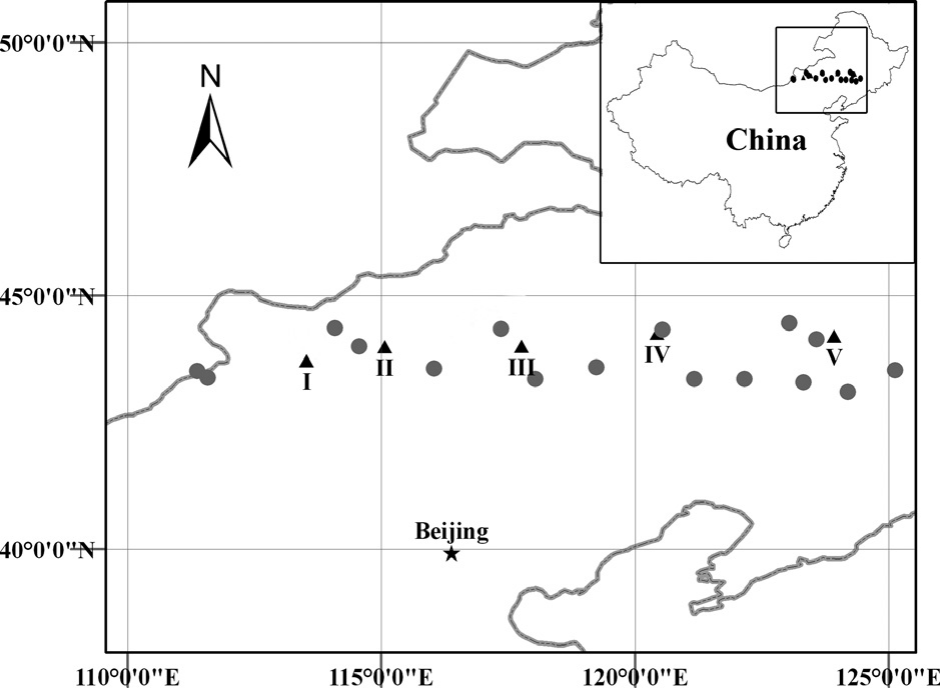
Locations of the controlled watering experiment sites conducted in 2011. The five sites are shown as closed black triangles and numbered I–V along the Northeast China Transect (NECT). The 16 meteorological stations along the NECT are shown as closed gray circles.

**Figure 2:**
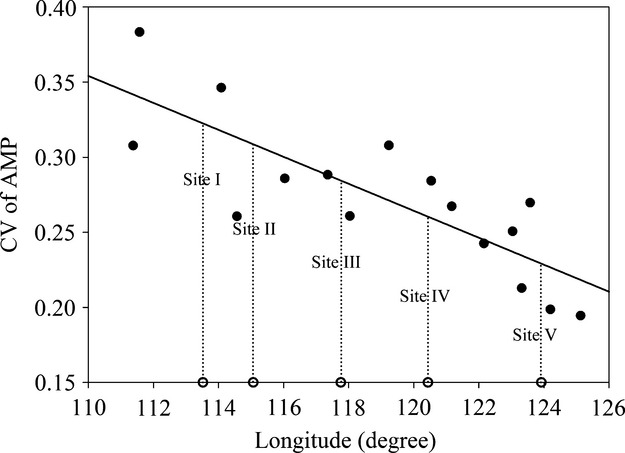
Coefficient of variation (CV) in mean annual precipitation of the meteorological stations along the Northeast China Transect (NECT).

The Northeast China Transect (NECT) runs in parallel to 43°30′N and ranges from 42° to 46°N and from 106° to 134°E, with little variation in the mean annual temperature (0–6°C), but a major variation in the annual precipitation (130–900 mm). There is a steady trend of decreasing stature, density, and foliage projective cover toward the dry end of this transect, with trees largely confined to the wet end (Ni and Zhang 2000; Ni and Wang 2004; Prentice et al. 2011). Five sites were selected from west to east along the NECT in 2011 and were defined as Site I (43°43.222′N, 113°31.629′E), Site II (43°59.726′N, 115°04.279′E), Site III (44°00.411′N, 117°45.856′E), Site IV (44°15.518′N, 120°26.365′E), and Site V (44°12.053′N, 123°55.519′E; Fig. 1 and Table 1). Site I was located in the desert steppes; Sites II, III, and IV were located in the “typical” steppes; Site V was located in the meadow steppes. Although Sites II and IV were also located in the “typical” steppes region, Site II was adjacent to the eastern edge of the desert steppes and Site IV was adjacent to the western edge of the meadow steppes (Fig. 1). The coefficient of variation (CV) in mean annual precipitation (MAP) becomes progressively lower from west to east along the NECT (Fig. 2 and Fig. S1). Furthermore, the site with greater precipitation variability is also drier. There are substantial interannual fluctuations and intra-annual changes in precipitation among the five sites (Fig. 2, Fig. S1 and Table 1). Thus, five controlled watering experiments were conducted at these sites, as they had ideal properties for our study.

**Table 1:** Characteristics of the sites from the Northeast China Transect (NECT) used in this analysis.

Site no.	Latitude (degree)	Longitude (degree)	Elevation (m)	Vegetation types	Temperature in 2011 (°C)	Precipitation in 2011 (mm)	MAP (mm)	CV of MAP	Species sampled	Species life forms
I	43.72	113.53	1027	Desert steppe	3.85	176.32	215.13	0.32	*Leymus chinensis*	Perennial grasses
									*Stipa krylovii*	Perennial grasses
									*Convolvulus ammannii*	Perennial forbs
II	43.99	115.07	1160	Steppe	3.82	220.82	262.03	0.31	*L. chinensis*	Perennial grasses
									*S. krylovii*	Perennial grasses
									*Artemisia pectinata*	Annual forbs
									*Artemisia frigida*	Perennial forbs
									*Allium polyrhizum*	Perennial forbs
III	44.01	117.76	1251	Steppe	3.92	304.37	362.88	0.28	*L. chinensis*	Perennial grasses
									*S. krylovii*	Perennial grasses
IV	44.26	120.44	381	Steppe	4.03	379.49	361.01	0.26	*L. chinensis*	Perennial grasses
									*Stipa grandis*	Perennial grasses
									*A. frigida*	Perennial forbs
									*Lespedeza bicolor*	Shrubs
									*Dracocephalum moldavica*	Annual forbs
V	44.20	123.93	178	Meadow steppe	4.33	471.69	475.21	0.23	*L. chinensis*	Perennial forbs
									*S. grandis*	Perennial grasses

MAT, mean annual atmospheric temperature; MAP, mean annual precipitation; CV, coefficient of variation in MAP was calculated using data obtained from 16 meteorological stations along the NECT (Table S1). MAP is the mean of the period from 1 January 1953 to 31 December 2003.

### Controlled watering experiment

At each site, 18 plots (1 m × 1 m) were set up and six levels of watering treatment (i.e., 0%, 20%, 40%, 60%, 80%, and 100% of local MAP) were assigned to the 18 plots in groups, with three replicates for each level. The MAP of the sites was obtained by linear interpolation on the basis of the meteorological stations along the NECT (Table S1). The water used for treatment is groundwater. Groundwater was divided into five equal parts, and evenly applied five times during the growing season, from June 18 to August 7. At each time, water was applied evenly to each plot using a portable 1 m^2^ plot boundary constructed of mild steel and a watering can, as a simple rainfall simulator. Some soil was piled up around the metal frame to minimize any leakage from the plot.

After treatment for one season, the mature leaves of all plants for the most common species were cut with scissors in late August 2011. Three species were harvested from Site I (*Leymus chinensis, Stipa krylovii,* and *Convolvulus ammannii*); five from Site II (*L. chinensis, S. krylovii, Artemisia pectinata, Artemisia frigida,* and *Allium polyrhizum*); two from Site III (*L. chinensis* and *S. krylovii*); five from Site IV (*L. chinensis, Stipa grandis, A. frigida, Lespedeza bicolor,* and *Dracocephalum moldavica*); and two from Site V (*L. chinensis* and *S. grandis*; Table 1).

### Carbon isotope measurement

All plant material was dried at 65°C for 48 h and ground to a fine powder by using a ball mill (MM400; Fa.Retsch, Haan, Germany). Aliquots (2.5 mg) of plant materials were weighed into tin capsules to analyze *δ*^13^C content using continuous-flow gas isotope ratio mass spectrometry (CF-IRMS) with Flash EA1112 and interface of Conflo III (MAT 253, Finnigan MAT, Germany). Pee Dee Belemnite (PDB) was used as the reference standard for C isotopic analyses. The standard deviation of repeated measurements of laboratory standards was ±0.15‰.

### Data analysis

All statistical analyses were performed using R 2.12.0 (R Development Core Team 2010). Correlation between foliar *δ*^13^C and precipitation was tested using linear regression. Differences between regression slopes were tested using Standardized Major Axis Tests ' Routines (SMATR), a freely available program (Falster et al. 2006). Two-way analysis of variance (ANOVA) was used to test the effects of species and precipitation on foliar *δ*^13^C values of each site (intrasite effects), as well as the effects of site and precipitation for common species among the sites (intersite effects).

## Results

### Intrasite differences in foliar *δ*^13^C values

**Figure 3:**
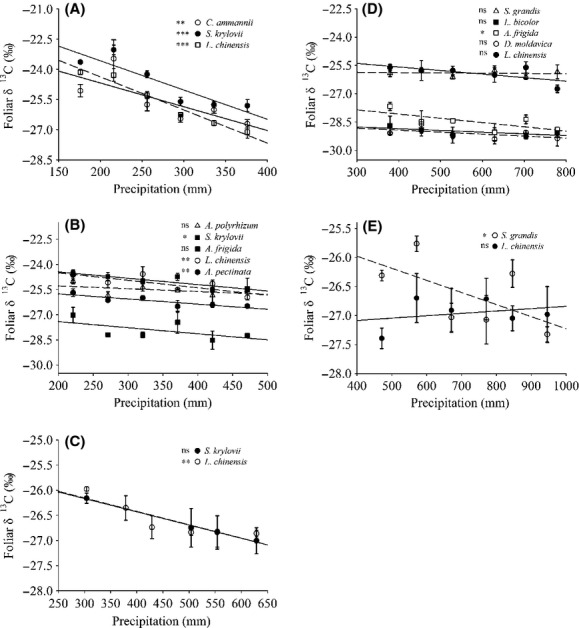
Response pattern of foliar *δ*^13^C to precipitation in common species at each site. (A–E) represent the pattern in common species at different sites. (A) Site I: *Convolvulus ammannii, Stipa krylovii, Leymus chinensis;* (B) Site II: *Allium polyrhizum, S. krylovii*, *Artemisia frigida*, *L. chinensis*, *Artemisia pectinata;* (C) Site III:*S. krylovii*,*L. chinensis;* (D) Site IV:*Stipa grandis*,*Lespedeza bicolor*,*A. frigida*,*Dracocephalum moldavica*,*L. chinensis;* (E) Site V: *S. grandis*,*L. chinensis*. The precipitation shown in these figures is equal to the sum of the local mean annual precipitation plus the amount of water applied to each plot. Each point is the mean of foliar *δ*^13^C ± 1SE. Where a point has no error bars, it is a missing value. Asterisks indicate the levels of significance, and “ns” stands for “not significant” levels.

Foliar *δ*^13^C values decreased with increasing precipitation for most species at each site (Fig. 3 and Fig. S2). Precipitation was strongly correlated with foliar *δ*^13^C for most species at each site (Fig. 3). Tests for homogeneity of regressions showed no statistical significant difference between the slopes of species at each site (Site I, *P *=* *0.93; Site II, *P *=* *0.29; Site III, *P *=* *0.60; Site IV, *P *=* *0.31; Site V, *P *=* *0.60; Fig. 3).

Two-way ANOVA revealed that both species and precipitation had significant effects on foliar *δ*^13^C among species (Table 2), except at Site III, which showed significant effects only for precipitation (Table 2). However, the interaction effect between species and precipitation on the variance of foliar *δ*^13^C showed no significant difference, except at Site V (Table 2).

**Table 2:** Two-way analysis of variance for species versus precipitation effects between species at each site.

Source	*P*-value				
	Site I	Site II	Site III	Site IV	Site V
Species	3.8e^−^^6^***	<2.2e^−16^***	0.40	<2.2e^−16^***	0.063
Precipitation	2.6e^−14^***	4.2e^−04^***	0.028*	0.034*	0.052
Species × Precipitation	0.20	0.61	0.94	0.56	0.049*

Asterisks indicate the level of significance:

*** 0.001

** 0.01

* 0.05.

### Intersite differences in foliar *δ*^13^C values

**Figure 4:**
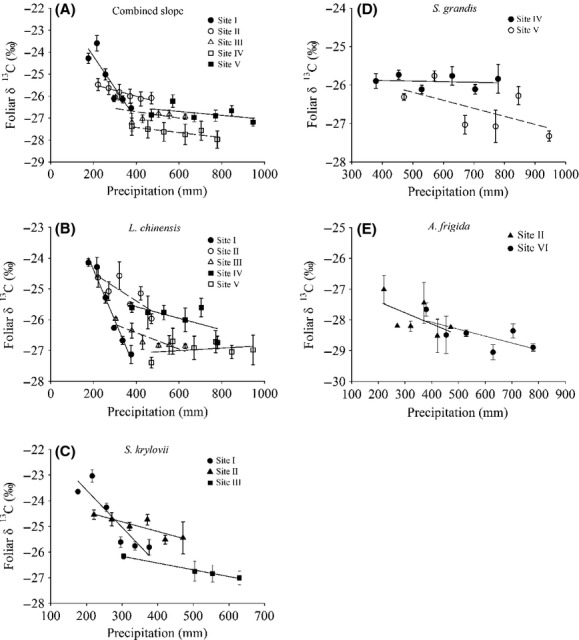
Response pattern of foliar *δ*^13^C to precipitation between sites for common species at different sites. (A) Combined slope for all species of each site. (B–E) Patterns of common species collected from different sites: (B), *Leymus chinensis*; (C), *Stipa krylovii;* (D), *Stipa grandis*; (E), *Artemisia frigida*. The precipitation shown in these figures is equal to the sum of the local mean annual precipitation plus the amount of water applied to each plot. Each point is the mean of foliar *δ*^13^C ± 1SE. Where a point has no error bars, it is a missing value.

No significant difference was found between all the slopes of species at each site, and thus, they were combined to form 1 slope for species (Fig. 4A). Negative relationship was found for each slope (Site I, *P *=* *9.0e^−12^; Site II, *P *=* *0.03: Site III, *P *=* *2.0e^−4^; Site IV, *P *=* *0.33; Site V, *P *=* *0.21; Fig. 4A). However, a strong statistically significant difference was found in the tests for homogeneity of these slopes (*P *=* *0.00; Fig. 4A). A similar pattern was also found in the common species found at different sites in the experiment (*L. chinensis*: *P *=* *9.1e^−12^, Fig. 4B; *S. krylovii*:*P *=* *2.6e^−5^, Fig. 4C; *S. grandis*:*P *=* *0.14, Fig. 4D; *A. frigida*:*P *=* *0.03, Fig 4E). Further, the slope of the line progressively decreased from Site I to Site V for all species in this study (Fig. 4).

Two-way ANOVA indicated that both site and precipitation had significant effects on *L. chinensis*,*S. krylovii, S. grandis,* and *A. frigida* (Table 3). Furthermore, the interaction effect between site and precipitation on the variance of *δ*^13^C also reached a strong significant level in *L. chinensis*,*S. krylovii,* and *S. grandis* (Table 3).

**Table 3:** Two-way analysis of variance for site versus precipitation effects in *Leymus chinensis*,*Stipa krylovii,* and *Stipa grandis*.

Sources	*P*-value			
	*L. chinensis*	*S. krylovii*	*S. grandis*	*Artemisia frigida*
Site	<2.2e^−16^***	3.9e^−12^***	1.1e^−05^***	0.02*
Precipitation	3.8e^−09^***	1.2e^−07^***	8.2e^−3^**	0.02*
Site × Precipitation	1.7e^−5^***	1.6e^−03^**	0.01**	0.50

Asterisks indicate the levels of significance:

*** 0.001

*** 0.01

* 0.05.

## Discussion

The foliar *δ*^13^C value tended to decrease as precipitation availability increased (Figs. 3,4), which is in agreement with the findings of other studies (Chen et al. 2002; Prentice et al. 2011; Wang et al. 2012). This study compared foliar *δ*^13^C values of species under different precipitation conditions, where the slope indicates the level of plasticity (i.e., a steeper line is considered to be more plastic; De Jong 1990). We hypothesized that the level of plasticity in foliar *δ*^13^C for species growing in the same habitat was apparently consistent (Fig. 3) and tests for homogeneity of regressions confirmed this hypothesis. Furthermore, the interaction effect between species and precipitation on the variance of foliar *δ*^13^C at the first four sites as revealed by the two-way ANOVA results suggested that there was no intrasite difference in plasticity (Table 2). For Site V, however, the difference in foliar *δ*^13^C between species was almost significant (Table 2), but the individual values were scattered and did not produce a significant effect (Fig. 3). Therefore, we attribute this finding to experimental error and conclude that the levels of plasticity are similar for species growing in the same habitat.

As expected, different levels of plastic populations were found at the five controlled experiment sites (Fig. 4). In the present study, when all the slopes at each site were combined to form one slope for all species (Fig. 4A), a strong statistically significant difference was found among the sites (*P *=* *0.00), that is, there was a clear species habitat influence on the levels of plasticity in foliar *δ*^13^C (Fig. 4A). The significant interaction effect between site and precipitation on the variance of foliar *δ*^13^C shown by two-way ANOVA suggested that there were different levels of plasticity in foliar *δ*^13^C in species growing at different sites or habitats (Table 3). Furthermore, it is worth noting that the same species growing at different sites – *L. chinensis* (Fig. 4B), *S. krylovii* (Fig. 4C), *S. grandis* (Fig. 4D) and *A. frigida* (Fig. 4E) – exhibited different levels of plasticity in foliar *δ*^13^C. The comparison of plasticity in foliar *δ*^13^C among different habitats for the same species showed a strong significant difference. This suggests that populations of the same species experiencing different environment conditions in their distribution exhibit different levels of plasticity in foliar *δ*^13^C. Liu et al. (2013) reported a negative relationship between foliar *δ*^13^C and precipitation for *L. chinensis* at a site over time, but a nonlinear response pattern at a large spatial scale. These findings are consistent with those of this study and could be explained by the different levels of foliar *δ*^13^C response patterns to precipitation across different sites for *L. chinensis*.

On the other hand, the level of plasticity in foliar *δ*^13^C was found to decrease from Site I to Site V (Fig. 4). Temporal environmental variation across the five sites might be reason for this difference of plasticity in foliar *δ*^13^C, because species growing in a more variable habitat are expected to have a high degree of plasticity than those growing in relatively a stable environment (Bazzaz 1991; Agrawal 2001; Pfennig and Murphy 2002; Hassall et al. 2005; Richards et al. 2006). In this study, the CV of MAP progressively decreased from Site I to Site V (Fig. 2 and Table S1); highly variable environments are thought to favor strong plasticity (Bazzaz 1991). However, the site with greater precipitation variability (west site along the NECT) was also drier. Thus, the influence of overall precipitation amount also might be another reason for the difference of plasticity in foliar *δ*^13^C. Drought stress caused by low precipitation can lead to stomatal closure and decreased C_i_/C_a_ (Stewart et al. 1995), eventually leading to increases in *δ*^13^C. Therefore, the apparent stomatal limitation at Site I would decline rapidly as water availability increases, whereas foliar *δ*^13^C with lower plasticity at Site V might have been caused because watering exceeded the threshold value of MAP above which foliar *δ*^13^C shows no significant change (Leffler and Enquist 2002; Wang et al. 2012). Furthermore, numerous studies have indicated that soil water availability is a determinant of foliar *δ*^13^C value for plants grown in a dry habitat (Ehleringer 1993; Schulze et al. 1996; Chen et al. 2007); however, Hanba et al. (2010) reported that soil water availability had a less conspicuous effect on foliar *δ*^13^C in C_3_ *Pocaeae* species grown in a humid climate in Japan. This suggests that the habitat-specific differences in foliar *δ*^13^C plasticity patterns found in this research are consistent with those reported previously. Plants grown at Site I showed sensitive response in foliar *δ*^13^C to precipitation; this could be because water is a determinant of foliar *δ*^13^C under dry conditions. On the other hand, plants grown at Site V did not show higher degree plasticity in foliar *δ*^13^C because of the lower importance of soil water availability in the humid environment.

In conclusion, this study shows that there was no significant difference in the levels of plasticity in foliar *δ*^13^C for the species growing in the same habitat in the temperate steppes, but a strong significant difference was found among different habitats for these species. This indicates that species' foliar *δ*^13^C response to precipitation is conservative at the same site due to their long-term acclimation, but the mechanism responsible behind this needs further investigations.

## Supporting Information

**Figure S1.** The seasonal precipitation and temperature pattern for each site in the controlled watering experiment conducted in 2011. **Figure S2.** Response pattern of foliar δ^13^C to precipitation in all species in the controlled watering experiment conducted in 2011. The precipitation shown in these figures is equal to the sum of the local mean annual precipitation(MAP) plus the amount of water applied to each plot. **Table S1.** Coefficient of variation (CV) of mean annual precipitation (MAP) of 16 meteorological stations along the Northeast China Transect (NECT).
