# Supplementary material for: Habitat-specific differences in plasticity of foliar δ13C in temperate steppe grasses
Source: Ecol Evol. 2014 Feb 12;4(5):648–55. doi: 10.1002/ece3.970 (PMC4098143; doi:10.1002/ece3.970)
Supplement: Supplementary file 1 — Figure S1. The seasonal precipitation and temperature pattern for each site in the controlled watering experiment conducted in 2011. Figure S2. Response pattern of foliar δ13C to precipitation in all species in the controlled watering experiment conducted in 2011. The precipitation shown in these figures is equal to the sum of the local mean annual precipitation(MAP) plus the amount of water applied to each plot. Table S1. Coefficient of variation (CV) of mean annual precipitation (MAP) of 16 meteorological stations along the Northeast China Transect (NECT). [file ece30004-0648-sd1.pdf]

Table S1. Coefficient of variation (CV) of mean annual precipitation (MAP) of 16 meteorological stations along the NECT

Site no.	Latitude (degree)	Longitude (degree)	Elevation (m)	MAP(mm)	SD	CV
1	43°31.20′N	111°22.80′E	1036.7	187.13	57.58	0.308
2	43°23.40′N	111°34.80′E	964.7	137.06	52.52	0.383
3	44°22.20′N	114°05.40′E	1181.6	224.57	77.74	0.346
4	44°00.60′N	114°34.20′E	1126.1	242.60	63.21	0.261
5	43°34.20′N	116°02.40′E	989.5	282.36	80.69	0.286
6	44°21.00′N	117°21.60′E	995.9	338.14	97.45	0.288
7	43°21.60′N	118°02.40′E	799.0	386.64	100.8	0.261
8	43°35.40′N	119°14.40′E	484.4	387.90	119.3	0.308
9	44°20.40′N	120°32.40′E	265.0	384.70	109.3	0.284
10	43°21.60′N	121°10.20′E	241.0	333.84	89.20	0.267
11	43°21.60′N	122°09.60′E	178.5	388.10	94.11	0.242
12	44°28.20′N	123°02.40′E	149.5	398.91	99.95	0.251
13	43°18.00′N	123°19.20′E	114.9	465.06	98.95	0.213
14	44°09.00′N	123°34.80′E	189.3	448.77	121.0	0.270
15	43°06.60′N	124°01.20′E	164.2	638.71	126.8	0.199
16	43°32.40′N	125°07.80′E	236.8	580.92	112.9	0.194

Note: Annual precipitation is the mean of the period from January 1, 1953 to December 31, 2003.

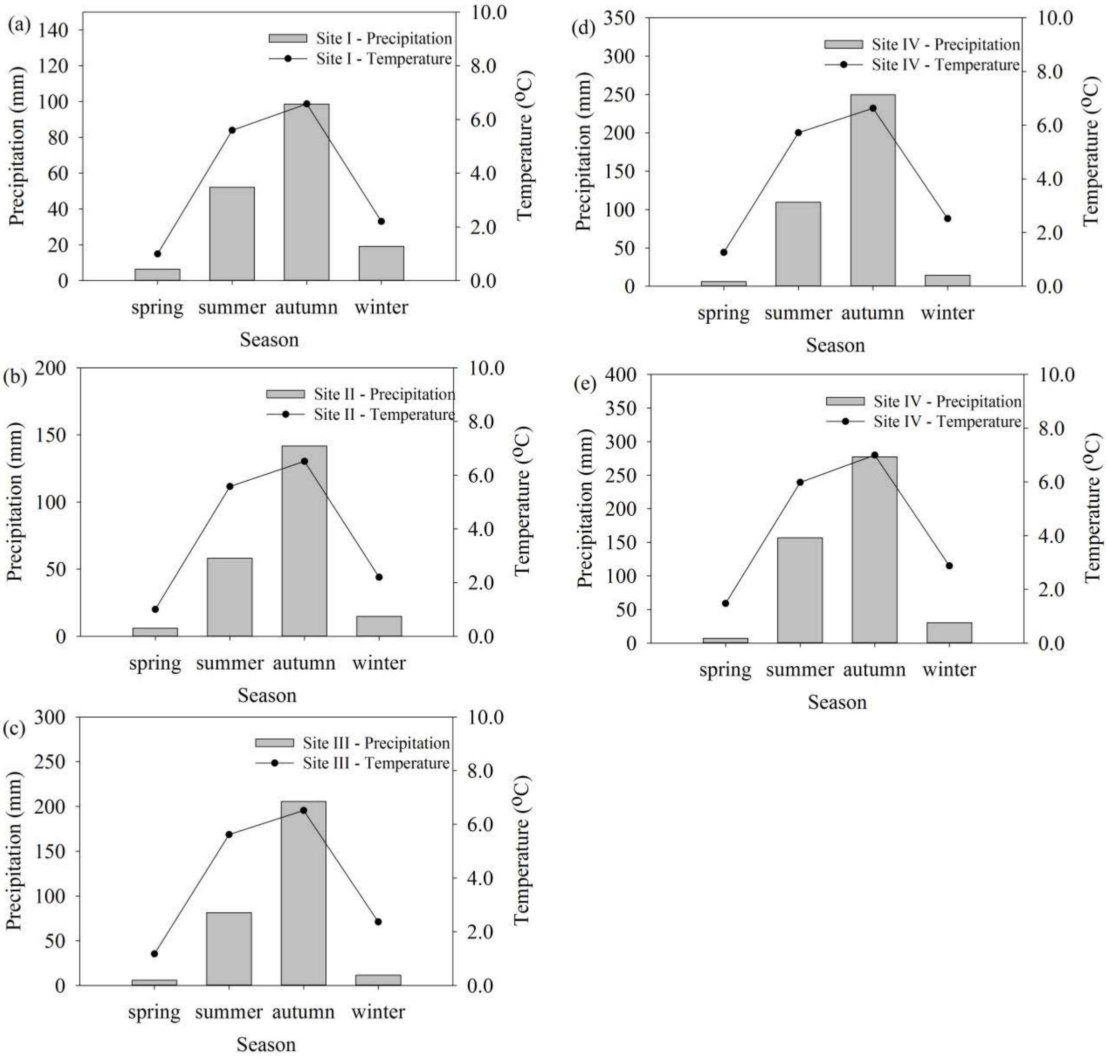

Figure S1. The seasonal precipitation and temperature pattern for each site in the controlled watering experiment in 2011.

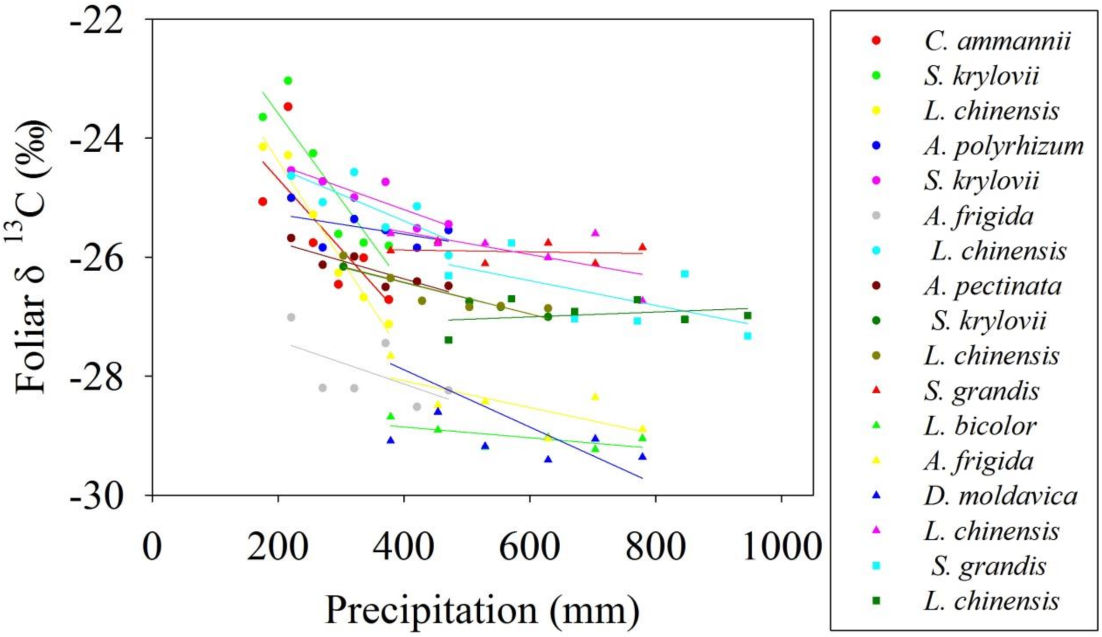

Figure S2. Response pattern of foliar $\delta^{13}C$ to precipitation in all species in the controlled watering experiment conducted in 2011. The precipitation shown in these figures is equal to the sum of the local MAP plus the amount of water applied to each plot.
